# Overview of Oral Potentially Malignant Disorders: From Risk Factors to Specific Therapies

**DOI:** 10.3390/cancers13153696

**Published:** 2021-07-23

**Authors:** Luigi Lorini, Coro Bescós Atín, Selvam Thavaraj, Urs Müller-Richter, Margarita Alberola Ferranti, Jorge Pamias Romero, Manel Sáez Barba, Alba de Pablo García-Cuenca, Irene Braña García, Paolo Bossi, Paolo Nuciforo, Sara Simonetti

**Affiliations:** 1 Medical Oncology Unit, Department of Medical and Surgical Specialties, Radiological Sciences and Public Health, ASST Spedali Civili of Brescia, University of Brescia, 25123 Brescia, Italy; luigilorini91@gmail.com (L.L.); paolo.bossi@unibs.it (P.B.); 2 Oral and Maxillofacial Department, Vall d’Hebron University Hospital, 08035 Barcelons, Spain; cbescos@vhebron.net (C.B.A.); jpamias@comb.cat (J.P.R.); masaezbcn@gmail.com (M.S.B.); vilwarin33@hotmail.com (A.d.P.G.-C.); 3 Head and Neck Pathology, Guy’s and St Thomas’ NHS Foundation Trust, London SE1 9RS, UK; selvam.thavaraj@kcl.ac.uk; 4 Comprehensive Cancer Center and Department of Oral and Maxillofacial Plastic Surgery, University Hospital of Würzburg, 97070 Würzburg, Germany and Bavarian Centre for Cancer Research, 97070 Würzburg, Germany; mueller_U2@ukw.de; 5 Department of Pathology, Vall d’Hebron University Hospital, 08035 Barcelona, Spain; malberola@vhebron.net; 6 Department of Medical Oncology, Vall d’Hebron University Hospital, Vall d’Hebron Institute of Oncology (VHIO), 08035 Barcelona, Spain; ibrana@vhio.net; 7 Molecular Oncology Laboratory, Vall d’Hebron Institute of Oncology (VHIO), 08035 Barcelona, Spain; pnuciforo@vhio.net

**Keywords:** oral potentially malignant disorders, risk factors and etiology, morphological features, molecular alterations, treatment

## Abstract

**Simple Summary:**

Oral potentially malignant disorders (OPMDs) include a group of oral mucosal diseases with different morphological characteristics that are able to progress to oral squamous cell carcinoma (OSCC). Given OSCC’s poor prognosis and high mortality, early diagnosis is a priority step in OSCC. Extrinsic and intrinsic risk factors and etiologies are involved in developing and malignant transformation of oral lesions, and different molecular alterations have been described in early lesions associated with a potential malignant behavior. This review summarizes the information about clinical, morphological and molecular features of OPMDs, with an emphasis on the early detection techniques and an overview of the surgical and systemic therapies’ effectiveness.

**Abstract:**

Oral squamous cell carcinoma (OSCC) is a very aggressive cancer, representing one of the most common malignancies worldwide. Oral potentially malignant disorders (OPMDs) regroup a variegate set of different histological lesions, characterized by the potential capacity to transform in OSCC. Most of the risk factors associated with OSCC are present also in OPMDs’ development; however, the molecular mechanisms and steps of malignant transformation are still unknown. Treatment of OSCC, including surgery, systemic therapy and radiotherapy (alone or in combination), has suffered a dramatic change in last years, especially with the introduction of immunotherapy. However, most cases are diagnosed during the advanced stage of the disease, decreasing drastically the survival rate of the patients. Hence, early diagnosis of premalignant conditions (OPMDs) is a priority in oral cancer, as well as a massive education about risk factors, the understanding of mechanisms involved in malignant progression and the development of specific and more efficient therapies. The aim of this article is to review epidemiological, clinical, morphological and molecular features of OPMDs, with the purpose to lay the foundation for an exhaustive comprehension of these lesions and their ability of malignant transformation and for the development of more effective and personalized treatments.

## 1. Introduction

Oral potentially malignant disorders (OPMDs) are defined as a group of oral mucosal lesions with an increased risk of malignant transformation. These disorders include a mixture of diseases with different clinical appearance, histological subtypes and risk factors/etiologies and comprise various entities, such as leukoplakia, erythroplakia, erythroleukoplakia, oral lichen planus, oral submucous fibrosis (OSF) and oral dysplasia [1,2]. Oral squamous cell carcinoma (OSCC) is one of the most frequent neoplasms worldwide, especially in countries with low- or middle-income economies, showing very aggressive behavior, propensity for lymph-node metastasis and bad prognosis [3]. Overall 5-year survival rates are as low as 40%; however, if diagnosed in the early stages (I and II), survival rates can exceed 80%. Hence, early detection, diagnosis and treatment of premalignant lesions is mandatory to prevent their transformation to OSCC and to increasing the 5-year survival rate [4].

Different risk factors have been associated to these lesions, most of them shared with OSCC, but the mechanisms and the causes of malignant transformations remain unclear. Lifestyle factors, such as use of tobacco, consumption of alcohol, derivatives of betel nut and sexually transmitted infection of human papilloma virus (HPV, mainly type 16) represent the main causes associated with OPMDs and oral cancer. Moreover, chronic mucosal inflammation and oral mucosal trauma from teeth and prosthetic devices have received increasing attention in several clinical and scientific studies [5]. Other suggested factors in common between oral cancer and premalignant lesions are alteration of the microbiome, systemic sclerosis, genetic disease with dysregulation of DNA metabolism (Zinsser–Engman–Cole syndrome, Fanconi anemia and Xeroderma pigmantosum), hematinic and micronutrient deficiency [6,7,8,9].

Despite how risk factors are widely accepted in the generation of OPMDs, no specific molecular alterations seem to be directly involved in malignant transformation of these lesions. Similar to other tumor models, oral cancer progression is driven by the accumulation of sequential molecular mutations that lead to premalignant changes as a first step and evolution in OSCC as the last station. Different mechanisms by which oral mucosa undergoes transformation in OSCC are reflected in varying degrees in premalignant lesions and include dysregulation of oncogenes or tumor suppressors (P53, MCM complex proteins), cytogenetic and epigenetic changes, and mitochondrial mutations [10,11].

People surveillance and early detection of OPMDs remain the most effective process to prevent cancer transformation, including regular dental examination and removal of implicated environmental and behavioral risk factors. Anyhow, prompt treatment of early stage disease may confer a significant survival benefit. For the management of OPMDs a variety of therapeutic strategies have been proposed and comprise electrosurgery, cryosurgery, surgical excision, CO2 laser and application of topical and systemic drugs (vitamin A, lycopene, retinoids, antibiotics and corticoids) [12,13]. A promising solution seems to be the photodynamic therapy (PDT), a non-invasive treatment that uses a combination of a photosensitizer (PS) and oxygen that activates reactive oxygen species (ROS), causing the death of microorganisms by apoptosis or necrosis [14,15]. However, there is still no evidence that treatment prevents malignant transformation, and, in some cases, counterproductive side effects have been found (e.g., scare formation, recurrence, etc.) [16].

This article aims to review the clinical, morphological and molecular features of OPMDs, with the purpose of understanding the processes related to malignant transformation and to update the information about early detection techniques and the efficacy of different therapeutic solutions.

## 2. Epidemiology and Risk Factors

### 2.1. Tobacco and Alcohol

Oral cancer represents a health problem worldwide due to its morbidity and mortality, with 5-year survival rates of about 50%, especially in developing countries and with death rate higher in male than in female [17]. Especially in the developing countries, mortality remains a challenging problem due to the lifestyle habits, the quality and the access to medical assistance, the health education of the population and the prevention of OPMDs. Actually, avoidable factors, such as tobacco and alcohol, are involved in 90% of cases with a synergic contribution [18,19,20]. It is established that the malignant transformation has as its starting point the normal mucosa exposed to various risk factors, with the progressive modification in different subtypes of OPMDs and, finally, the development of OSCC. Chronic exposure to alcohol and tobacco alters the genetic stability of oral mucosa keratinocytes, involving different genes such as NOTCH1, TP53, EGFR, CDKN2A and STAT3, found commonly in premalignant lesions and carcinomas [21,22]. Synergic mutagenic effects and an increase of oral mucosa permeability by alcohol and tobacco lead to the alteration of tumor and immune microenvironment, promoting cell adhesion loss and invasion [23].

### 2.2. HPV (Human Papilloma Virus)

HPV infection, especially by HPV-16 and HPV-18 oncogenic types, is recognized as a strong risk factor of OSCC, localized in the posterior tongue, tonsils and upper pharynx [24,25]. Therefore, it would be fair to expect a close association between oncogenic types of HPV and OPMDs. Nevertheless, current data demonstrate that the role of HPV infection is not yet clear in OPMDs and is involved in the multistep process to malignant transformation. In fact, in different cohorts of patients with oral leukoplakia, lichen planus and dysplasia, the detection rate of HPV is variable (from 4.8% to 40) [17,18,26]. However, though HPV oncogenic types have a higher prevalence in OPMDs than in the healthy oral mucosa, there is insufficient evidence of the malignant potential of the virus in premalignant lesions.

### 2.3. Areca Nut Chewing and Microbiome Alteration

Areca (betel) nut is one of the most consumed addictive substance worldwide, especially in Asia and Pacific area [27,28]. Areca nut contains pro-carcinogenic chemical components, such as copper or arecoline, often added to tobacco quid, and its chewing is considered an independent cause of oral cancer [29,30,31,32]. Exposure of oral mucosa to areca nut provokes various oral precancerous lesions and conditions, especially lichen planus and oral submucous fibrosis [33,34,35]. The mechanisms of oral carcinogenesis induced by areca nut remains poorly understood, but it has been demonstrated that regular chewing induces chronic inflammation, direct DNA damage, inhibition of tumor suppressor and repression of DNA repair or conversion of stem cells that damage epithelial cells of the oral mucosa [36,37]. All of these alterations seem to be related to a dysregulation of the oral microbiome with reduced levels of commensal bacteria critical to maintaining homeostasis. Elevated levels of *Streptococcus*, *Actinomyces* and periodontal pathogens such as *Fusobacterium* have been observed in current chewers who developed premalignant lesions and OSCC [6,38]. The mechanisms by which the oral microbiome alteration causes the mucosa lesions are still under study. However, it seems that the dysregulation of the immune microenvironment, due to the disruption in the normal oral microflora and the increase of pathogenic bacteria, is involved in oral tumorigenesis. Different species of bacteria are able to trigger cell proliferation and permanent genetic mutations by their products and metabolites and inducing proinflammatory cytokines’ production, inhibition of antitumoral immune cells, malignant transformation and cellular invasion [39,40].

### 2.4. Other Risk Factors

In addition to the most frequent risk factors, OPMDs development is associated with different uncommon disorders or micronutrients deficiencies [41]. Patients with autoimmune chronic diseases, such as scleroderma (systemic sclerosis), show high susceptibility to develop cancer compared with the general population, and there is some evidence that this may include the risk for OSCC, probably due to the chronic inflammation and immunosuppression of the diseases or treatment [42,43]. Some genetic syndromes that cause dysregulation of DNA metabolism of oral epithelium can lead to premalignant and malignant lesions in this region. Dyskeratosis congenita (Zinsser-Engman-Cole syndrome), Bloom syndrome (BS; congenital telangiectatic erythema), Xeroderma pigmentosum and Fanconi anemia show an increased incidence of OPMDs and OSCC [9,44].

## 3. Early Diagnosis

A few studies have focused on performing a systematic review and meta-analysis on how a late diagnosis might affect the prognosis, as well as the factors that may contribute to this late diagnosis. It has been studied how referral delay directly affects TNM at diagnosis and survival [45]. General practitioners’ educational competence in diagnosis is a key factor to detect such cases at an early stage, and improvement of their training at a primary-care level is necessary to achieve the goal of early diagnosis and prevention [46]. The study by Varela-Centelles highlighted that the factor that most delayed treatment was time lapse between the moment when patient presented symptoms for the first time and the moment when he/she decided to seek medical care [47]. Lack of knowledge of the disease and its presentation by the most vulnerable patients who are at high risk of developing oral cancer plays a major role, and the study sheds light on the importance of developing campaigns explaining symptoms and risk factors on oral cancer, specially targeting high-risk groups in the population.

Still, today, the gold standard for the diagnosis of oral lesions is the visual exploration of the oral cavity and tissue biopsy of suspicious lesions. Other adjunctive techniques have been studied, such as oral cytology, salivary biomarkers, light-based techniques (auto-fluorescence, chemiluminescence, spectroscopy, etc.) and tissue staining. When a lesion cannot be clinically identified as benign or malignant, these adjunctive tests may help deciding whether a biopsy is required or not. This may help to reduce hospital referrals and the number of biopsies required. The evidence on the role of these adjunctive tests, when there is a clinically evident lesion, has been studied in two systematic reviews and meta-analyses. The first was published by Fuller et al. [48], and they concluded that oral cytology holds a higher diagnostic value than a specialist’s oral examination, which holds a higher value than in vivo toluidine blue staining. The cost of cytology and the fact that it may delay final tissue diagnosis with biopsy should be taken into account. They also stated that conventional cytology was more specific than other more complex cytology techniques, such as computer-based cytology or liquid cytology. Spectroscopy had the highest sensitivity and accuracy, but limitations may arise in certain anatomic locations, and the higher cost should be taken into consideration. The other review article published by Macey et al. [49] also showed that, even if none of the tests could replace tissue biopsy, oral cytology was the adjunctive test with the highest potential, with a higher specificity when compared to staining or light-based detection techniques. It must be stressed that, in their review, they could not include any studies on the accuracy of blood or saliva tests because these studies were not eligible for inclusion. They concluded that the level of evidence is low and that there are no studies conducted in the primary-care setting, so their application in this scenario is unknown.

Another focus to study is the role of screening for oral cancer. The Cochrane reviews published in 2013 [50,51] showed that oral-cancer screening did not decrease mortality in the general population, but it did reduce mortality by 24% in the high-risk population (alcohol- and tobacco-users). It decreased diagnosis at an advanced stage and 5-year survival in the general population. Hence, the visual examination of the oral cavity decreased mortality in a certain high-risk group of patients, and there is no evidence that the use of other adjunctive tests would benefit the screening. In the meantime, an opportunistic systematic exploration of the oral cavity of the population performed by general practitioners in primary-care settings is recommended, especially of high-risk patients. Up until today, there is no evidence on whether an oral cancer and pre-cancer screening programs in Europe would benefit the asymptomatic population due to limitations on the evidence published in this setting, and future studies will be required to answer this question [52].

## 4. Clinicopathological Features of OPMDs

**Figure 1:**
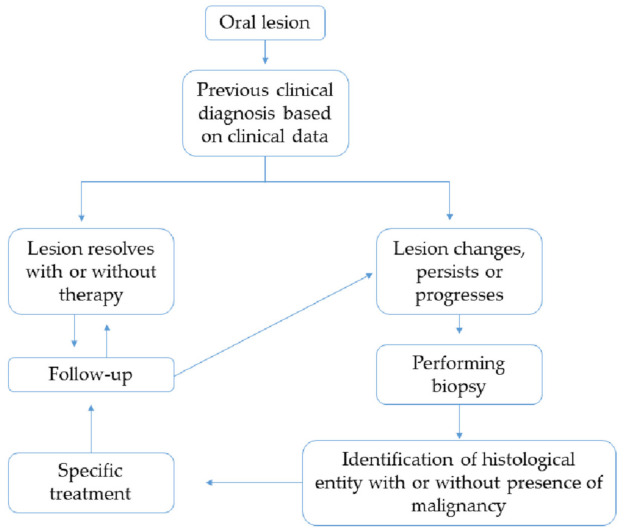
Flowchart representing the steps in identification, diagnosis and managing of OPMDs.

The definition of OPMDs is above all a clinical description, but morphological confirmation is the most important procedure, not only to identify the correct lesion. In the flowchart represented in Figure 1, a previous diagnosis is made based on clinical data of the patient. In cases where the lesions persist, change or progress, biopsy is mandatory, and histological examination represents the most complete and useful tool to exclude or confirm the presence of dysplasia in order to assess the risk of malignant transformation.

**Figure 2:**
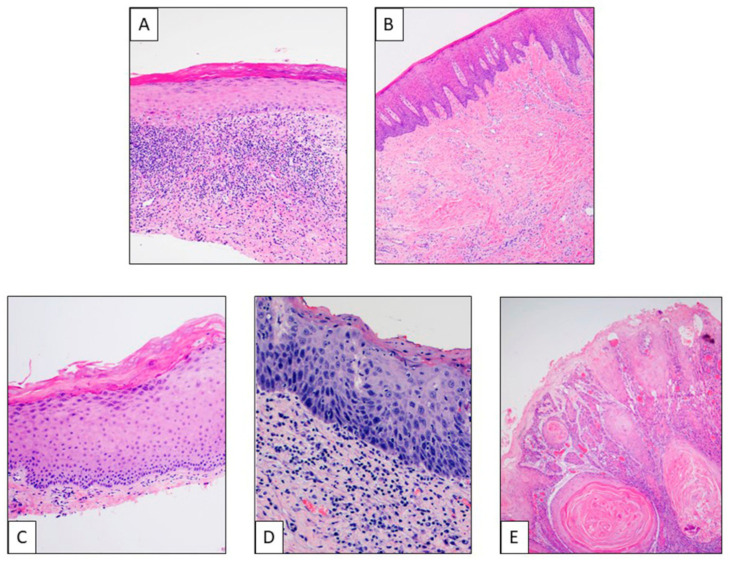
Different histological entities of oral lesions. (**A**) **Lichen planus**: Histologically characterized by degeneration of the basal cell line of the epithelium oral mucosa, with presence of diverse degrees of orthokeratosis and parakeratosis besides thickness of spinous layer and a characteristic band-like lymphocytic infiltration at the basal level and superficial submucosa. (**B**) **Oral submucous fibrosis**: This is a chronic progressive scarring lesion, morphologically showing evident submucosal changes such as fibrosis, diffuse chronic inflammatory infiltrate, atrophy of minor salivary glands, skeletal muscle atrophy, band-like infiltrate, edema and congestion, and vesicle formation. At mucosa level, we found changes such as atrophic changes, pigment incontinence, ulceration with granulation tissue, hyperplastic changes, dysplasia or malignant transformation. (**C**) **Low-grade dysplasia**. (**D**) **High-grade dysplasia/in situ carcinoma**: Dysplasia is a premalignant condition that refers to abnormal epithelial growth characterized by architectural and cytologic atypia, including dyskeratosis, basal cell hyperplasia and anaplasia, keratin pearls, etc. Low-grade refers to architectural and cytologic atypia affect from 1/3 to 2/3 of epithelium; high-grade/in situ carcinoma represents full thickness cytological or architectural atypia, without invasion of the neoplastic keratinocytes through the basement membrane. (**E**) **Infiltrating squamous cell carcinoma**: Malignant neoplasm characterized by severe dysplasia of the surface epithelium with invasion of the stromal–epithelial interface.

Different entities have been described, such as oral leukoplakia (OL), oral erythroplakia (OE), erythroleukoplakia, oral lichen planus (OLP), oral submucous fibrosis (OSF) and oral epithelial dysplasia (OED), each of them with diverse risks of malignant transformation (Figure 2). Clinicopathological features are described in Table 1.

### 4.1. Oral Leukoplakia (OL)

OL is a common and potentially malignant condition of the oral mucosa defined as “A white plaque of questionable risk having excluded (other) known diseases or disorders that carry no increased risk for cancer” [53]. OL is more frequent in men, and the associated risk factors are tobacco, areca nut, alcohol and HPV. Clinically, OL can be divided into two subtypes: homogeneous and non-homogeneous. Homogenous lesions appear as superficial and flat and non-homogenous ones are more irregular, verrucous or exophytic. Non-homogenous OL presents a higher risk for malignant transformation. Biopsy is a real useful tool to confirm the diagnosis with high specificity and accuracy. Prevention and treatment of OL include clinical surveillance and risk factors elimination as first step. However, the initial treatment can be made with bleomycin or retinoids. The complete response ratio was not high (10–27%), and the recurrence of OL was reported as being approximately 50% after treatment with topical retinoic acid [54]. The most common treatment options are surgical excision or CO_2_ laser therapy, together with cryotherapy and photodynamic therapy, especially in those case showing moderate and severe epithelial dysplasia. Efficacy of these treatments is high (70–90% complete response ratio), but the recurrence ratio is about 25–30% of patients [12,13].

### 4.2. Proliferative Verrucous Leucoplakia (PVL)

PVL represents an uncommon type of multifocal OL with aggressive behavior and resistance to therapies. More frequent in women after the sixth decade of life, it clinically shows an asymptomatic white and verrucous plaque. Morphologically, these lesions often present different grades of dysplasia and progress to OSCC. A close follow-up is mandatory [55].

### 4.3. Oral Erythroplakia (OE)

OE represents a single erythematous oral mucosal lesion with high malignant transformation rate [2]. Is it associated with tobacco and alcohol abuse and high-risk HPV, and its prevalence is between 0.02% and 0.83%, affecting adults of middle age [56]. Clinical appearance is characterized by a solitary erythematous flat lesion within the oral cavity. Histological diagnosis is of exclusion with differential diagnosis of other lesions [57]. Due to the high malignant transformation rate, early diagnosis and treatment are mandatory. Surgical excision and ablation by CO2 laser is the recommended therapy with low associated morbidity [58].

### 4.4. Oral Lichen Planus (OLP)

Lichen planus is a chronic autoimmune, inflammatory disease which may affect different organs, such as skin, oral or genital mucosa. Several factors have been proposed to be able to produce this kind of lesion, but the etiology remains unclear. OLP is more frequent in women in middle age, and the malignant transformation rate is low (<1–6%). Typically, lesions are localized in the buccal mucosa, bilaterally, and usually asymptomatic. Clinical aspect present different type of OLPs, such as reticular, plaque-like, bullous, atrophic or erosive. The most common is the reticular subtype characterized by thin white plaques called “Wickham’s striae”. Atrophic and erosive patterns are associated with significant pain. Bullous pattern is the least common type of OLP that is characterized by bullae formation. Plaque-like type is frequently observed in smokers. Lesions may have a combination of characteristics [59,60]. The diagnosis of OLP is typically made clinically and confirmed histologically. Treatment of OLP is generally palliative and not curative. The principal goal of management is to reduce inflammation and alleviate symptomatology with topical steroids [61,62].

### 4.5. Oral Submucous Fibrosis (OSF)

OSF is chronic disorder of the oral cavity characterized by collagen deposition and strong fibrosis, with high potential of malignant transformation (rate 7–30%) [63]. It is frequent in Asian populations and is especially associated with betel nuts chewing; however, the etiology seems to be multifactorial. Patients present symptoms, such as pain, xerostomia, a burning sensation, taste disorders and limitation in buccal motility. Biopsy is important to lead the correct diagnosis. Four histopathological stages are described based on the increase of fibrosis and chronic inflammation of oral submucosa, progressive atrophy of epithelium and degeneration of muscle fibers [64]. Recent evidences show that the malignant transformation could be caused by the alkaloids of areca nut that are capable of stimulating fibroblasts’ growth, the production of cytokines (especially involving the TGF-β signaling) and immune cells’ activation. These mechanisms provoke microenvironment alteration and cellular dysregulation of proliferation, survival, differentiation, DNA repair function and tumor transformation [65,66]. The current therapy options have the aim to reduce symptoms and to improve the patients’ quality of life. They include anti-inflammatory and anti-fibrosis drugs (steroids, IFN-γ, collagenase, etc.), physical treatment (mouth exercising devices) and in severe OSF surgery. However, there are no established guidelines for treatment for clinicians [66].

### 4.6. Oral Epithelial Dysplasia (OED)

While OPMD is ascribed to clinical presentations, oral epithelial dysplasia (OED) is histo-morphologically defined as a spectrum of epithelial changes associated with an increased risk of transformation to carcinoma. The World Health Organization (WHO) recognizes several morphological features of OED and classifies these as either “architectural” (disordered tissue organization) or “cytological” (individual cell abnormality) (Table 2) [67]. Grading of dysplasia is essential to inform subsequent management of any given OPMD. The overall evidence indicates a positive correlation between the likelihood and time to malignant transformation with increasing degrees of dysplasia. The most widely utilized grading system is that proposed by the WHO, which utilizes three-tiers (mild, moderate and severe dysplasia) [68]. This system takes into account, but is not limited to, the epithelial thickness in thirds affected by dysplastic changes. The WHO system is not entirely prescriptive, and the cutoff between each grade is poorly defined, further compounding the issue of suboptimal reproducibility. In an attempt to overcome this, the most recent edition of the WHO Classification of Head and Neck Tumors also tabled a binary system (high- versus low-grade) and suggested cutoff criteria between the grades [69,70]. Ultimately, however, the goal of any grading system is not reproducibility, but to inform clinical management within a multidisciplinary context. To this end, clear communication to the clinician of the pathologist’s intent in assigning a grade is paramount, regardless of the system used.

#### HPV-Associated Oral Epithelial Dysplasia

There is increasing recognition of a subset of OED characterized by distinct histo-morphological features attributable to high-risk types of HPV, mainly HPV-16 [71,72,73,74,75]. This entity is distinguished from conventional OED by the presence of karryorhexis, isolated suprabasal apoptotic keratinocytes, occasional nuclear molding and variable koilocyte-like cells in the superficial zone [71]. There is strong and diffuse immunoreactivity for p16, but the defining diagnostic criterion is the demonstration of transcriptionally active high-risk HPV, usually by RNA in situ hybridization [71,74]. HPV testing by consensus PCR alone, in the absence of the aforementioned morphologic changes, is insufficient for the diagnosis of HPV OED. Progression to carcinoma has been reported, but the overall malignant transformation rates currently remain unknown, since reports are limited to small case series. No accepted grading system is established; therefore, HPV OED should, for the present, be graded and clinically managed according to conventional criteria until the significance of these specific cumulative histological changes become known [76].

## 5. Molecular Alterations

Loss of heterozygosity (LOH) of certain chromosomal loci has a central role in OPMDs malignant transformation. Back in 1996, the presence of LOH of 9p21or 3p14 in a series of oral leukoplakia was described for the first time. LOH of at least one of these loci was present in 51% of cases; among them, 37% developed head and neck squamous cell carcinoma (HNSCC). These chromosomal loci encoded tumor-suppressor genes, critical in the process of carcinogenesis, such as tumor protein 53 (TP53) and Cyclin Dependent Kinase Inhibitor 2A (CDKN2A) [77]. LOH at 9p21 persisted regardless of complete or histologic response to chemo-preventive strategy with a regimen of 13-cis-retinoic acid, interferon alfa and α-tocopherol [78]. Subsequent series confirmed that 28% of OPMDs harbored LOH of 3p14, 9p21 and/or other loci. Patients with LOH had a risk of 35% of developing malignant transformation. This risk increased to 69% if the patient had a history of oral cancer [79].

A prospective cohort divided 296 patients affected by oral dysplasia in high risk and low risk of malignant transformation based on the presence of LOH in 3p and/or 9p: the high-risk cohort revealed a 22, 6-fold increased risk of malignant transformation. LOH on 9p had a higher association with malignant transformation when compared to LOH on 3p. LOH on 4q and 17p were also found to be important predictor of increase carcinogenesis, leading to a division of OPMDs according to malignant transformation probability in low risk (LOH of 17p or 4p), intermediate risk (LOH of 9p alone or associated with either LOH of 17p or 4q) and high risk (LOH of 9p, 4q and 17p) with a 5-year malignant transformation rate of 3.1, 16.3 and 63.1%, respectively [80].

In 2016, in the EPOC trial [81], OPMDs were defined at high risk if they presented LOH of 3p14 and/or 9p21 with a history of oral cancer or LOH of 3p14 and/or 9p21 and additional LOH on 17p or 8p or 11p or 4q or 13q. Results from this study confirmed the role of LOH as a negative prognostic factors for malignant transformation with a 3-year cancer-free survival of 74% for LOH-positive patients, compared with 87% for LOH-negative patients (HR 2,19; 95% CI 1.25–3.83; *p* = 0.01); moreover, increased epidermal growth factor (EGFR) copy number was associated with LOH profiles. Another chromosomal abnormality described in OPMDs is aneuploidy. In a meta-analysis, aneuploidy was associated with an increased risk in malignant progression [82,83].

There are other molecular alterations and biomarkers associated with OPMD in addition to LOH and aneuploidy, such as the overexpression of podoplanin and TP53 isoform delta (Np63), deregulation in phosphatidylinositol 3 kinase-protein kinase B (PI3KB) and fibroblast growth factor (FGF) pathway, increased epidermal growth factor (EGFR) copy number [84,85,86,87], changes in microRNA (miR) expression, and epigenetic modifications. High levels of miR-21, miR-345 and miR-181b and low levels of miR 142-5 are associated with malignant progression of OPMD; final stages of carcinogenesis may also involve changes in miR-196a and miR-206. Various reports support the role of these miRs in carcinogenesis due to the involvement in the regulation of target genes, such as TPM1, PTEN, and bcl-2 in the regulation of immune system [88,89,90,91,92,93]. Regarding epigenetic modifications, in some series, 40% of cases had hypermethylation of p16 inhibitor of cyclin-dependent kinase4A, and almost all of these cases evolved to squamous cell carcinoma [94]. Another common epigenetic alteration is 06-Methylguanine-DNA Methyltransferase (MGMT) methylation, which is present in 50–80% of OPMDs [95].

Various studies have analyzed the impact of immune infiltration in malignant transformation of OPMDs: increased T-cells CD4+ and CD8+ infiltration has a protective effects, while increased levels of programmed cell death protein 1 (PD-1) and programmed cell death protein ligand (PDL-1), decreased T-cells CD3+ and increased T-cells helper 1 infiltration promote cancer progression [96,97,98]. Increased inflammatory systemic activity has been found in patients with HNSCC and OPMDs compared with healthy control, however prognostic impact on malignant transformation has not been confirmed yet [57].

A recent transcriptomic classification of OPMDs has been generated integrating the microarray gene expression analysis from 86 OPMDs [99]. This classification defines six distinct clusters of disease based on main biological features and deregulated pathways, mirroring the pathways present in head and neck squamous cell carcinomas: defense response, immunoreactive, human papilloma virus related, classical, hypoxia and mesenchymal clusters. Whereas the immunoreactive pathway reduced the risk of cancer transformation, the hypoxia, mesenchymal and classical clusters were associated with an increased risk of malignant progression. Other report identifies two main OPMDs gene-expression subtype, immunological and classical one; the first is characterized by strong enrichment in immune-related pathways; while the second one is characterized by over- expression of Epidermal Growth Factor Receptor (EGFR) as well as a significant enrichment by EGFR signaling pathway. Interestingly these two different OPMDs subtypes had similar rate of malignant transformation [90].

## 6. Update of OPMDs Therapeutic Strategies

The treatment of OPMDs is a two-step process that starts with elimination of inflammatory conditions. [100]. If these measures fail, the second step is surgical/invasive diagnostics and treatment. Mainly CO2, Nd:YAG, Er:YAG and diode lasers are used, with the advantages of less bleeding, swelling and pain [101].Especially in high-grade lesions and of course in carcinoma in situ, a complete resection is the treatment of choice [102]. Photodynamic diagnosis (PDD) and therapy is used for detection, as well as treatment, of oral neoplasia and potentially malignant lesions. Especially in oral lichen planus (OLP), PDD might be a good treatment option [103,104,105]. PDD is also used as a therapy for (fungal) infections [106,107].

Although surgical resection remains the main treatment for OPMDs lesions frequently recur and develop malignant transformation. In addition, patients with OPMD are at increased risk of developing second cancers due to the field cancerization of oral cavity [108]. Hence, chemoprevention emerges as a strategy to reverse OPMDs’ malignant transformation.

Various agents have been studied, including anti-inflammatory drugs (such as celecoxib and indomethacin), drugs targeting p53 mutation (such as ONYX-015), antioxidant agents (such as retinoids, Bowman Birk inhibitors and green tea extract), drugs targeting peroxisome-proliferator-activated receptors gamma (PPAR-γ) (such as thiazolidinediones) and drugs targeting EGFR (such as erlotinib). However, none of these agents was able to gain enough evidence to be implemented into clinical practice [108,109,110,111]. The EPOC trial is the largest randomized study [81]. This study evaluated the activity of erlotinib in patient affected by high-risk OPMDs (defined as LOH on 3p14 and/or 9p21 with a history of oral cancer or LOH at 3p14 and/or 9p21 and additional LOH on 17p or 8p or 11p or 4q or 13q). Erlotinib did not achieve to improve on the primary endpoint, which was 3 years of cancer-free survival (CFS). Despite this negative result, this study is a landmark, as this study incorporated the most accepted OPMDs classification nowadays.

The two most promising therapies currently under evaluation are vandetanib and immune checkpoint inhibitors (ICIs). Accrual of the study of vandetanib on high-risk OPMDs was completed in 2018, and we are now awaiting for results (NCT01414426). On the other hand, the activity of ICIs in HNSCC has support the evaluation of ICIs in OPMDs [112,113,114]. Ongoing clinical trials are evaluating activity of avelumab (NCT04504552) and sintilimab (NCT04065737) in high-risk OPMDs (based on LOH 3p14 and/or 9p21) and pembrolizumab (NCT03603223) in OPMDs with moderate-to-severe dysplasia.

## 7. Conclusions

OPMDs represent a group of different clinical, morphological and molecular entities of oral mucosa, with an increased risk of malignant transformation. These lesions share common risk factors and molecular alterations, also frequently found also in OSCC. Elimination of causing risk factors, people surveillance and early diagnosis of OPMDs remain the most effective process to reduce OSCC development. However, important advances have been made in the fields of surgical and systemic treatment, with the use of laser vaporization and photodynamic therapy and the opening of several clinical trials with specific targeted therapies and immunotherapy.

## Figures and Tables

**Table 1:** Clinicopathological features and malignant potential of OPMD lesions.

OPMD Entity	Common Oral Sites	Clinical Appearance	Histopathology	Prevalence	Affected Population	Malignant Potential
**Leukoplakia**	Any mucosal site		Two distinct appearances may be seen as dysplastic or non-dysplastic leukoplakia	0.02%	Men >40 years	
Homogenous type		White, flat with clearly, evident borders				Low
Nonhomogeneous type (erythroleukoplakia)		White/red, verrucous/exophytic, or nodular presentation				High
**Proliferative verrucous leukoplakia**	Gingiva, buccal mucosa, alveolar ridges, tongue	Asymptomatic, nonhomogenous white plaque often with a verrucous, keratotic surface. Multifocal presentation	Ranging from single hyperkeratosis to verrucous hyperplasia and varying degrees of dysplasia	<1%	Women >60 years	High
**Erythroplakia**	Buccal mucosa, palate, ventral tongue, floor of the mouth	Asymptomatic, erythematous oral mucosal lesion with a smooth or velvety in appearance	Epithelium is nonkeratinized, thin, and atrophic, allowing for visualization of underlying microvasculature; squamous hyperplasia may be seen without concomitant dysplasia (benign), varying degrees of dysplasia, or carcinoma in situ	0.02% to 0.83%	Adults >45 years	Very high
**Lichen planus**	Buccal mucosa, followed by the gingiva (desquamative) and the tongue	Variable forms: reticular, papular, plaque, erythematous or atrophic, erosive, or bullous forms; a combination of characteristics of different subtypes may coexist	Band-like lymphocytic infiltration and liquefaction degeneration of the basal cell layer; other features include hyperkeratosis, the presence of civatte bodies, and hydropic degeneration of basal cells	0.5% to 2.2%	Women between 30 and 60 years	Low
**Oral submucous fubrosis**	Any mucosal site	Initial phase: burning sensation and/or intolerance to spicy food. It gradually to juxtaepitelial fibrosis of the oral cavity	Mucosal changes such as atrophic changes, pigment incontinence, ulceration with granulation tissue, hyperplastic changes, dysplasia, and carcinoma. Submucosal changes such as fibrosis, diffuse chronic inflammatory infiltrate, atrophy of minor salivary glands, skeletal muscle atrophy, bandlike infiltrate, edema and congestion, and vesicle formation	0.2–2.3% in males and 1.2–4.6% in females	Asians population with age range from 11 to 60 years	High

**Table 2:** Modified WHO morphologic criteria of OED. In addition, verrucous morphology, budding rete processes, spontaneous apoptosis, ortho- or para-keratosis with abrupt lateral demarcation, subepithelial lymphocytic infiltrate and bulky suprabasal proliferation are increasingly accepted features of OED.

Architectural Changes	Cytological Changes
Irregular stratification	Abnormal variation in nuclear size
Loss of polarity of basal cells	Abnormal variation in nuclear shape
Bulbous rete ridges	Abnormal variation in cell size
Increased number of mitotic figures	Abnormal variation in cell shape
Premature keratinization in a single cell	Increased nuclear/cytoplasm ratio
Squamous eddies within rete ridges	Atypical mitotic figures
Loss of intracellular cohesion	Increased number and size of nucleoli
	Hyperchromasia
